# Characterizing microscopic and submicroscopic malaria parasitaemia at three sites with varied transmission intensity in Uganda

**DOI:** 10.1186/s12936-016-1519-8

**Published:** 2016-09-15

**Authors:** John Rek, Shereen Katrak, Hannah Obasi, Patience Nayebare, Agaba Katureebe, Elijah Kakande, Emmanuel Arinaitwe, Joaniter I. Nankabirwa, Prasanna Jagannathan, Chris Drakeley, Sarah G. Staedke, David L. Smith, Teun Bousema, Moses Kamya, Philip J. Rosenthal, Grant Dorsey, Bryan Greenhouse

**Affiliations:** 1 Infectious Diseases Research Collaboration, Kampala, Uganda; 2 Department of Medicine, University of California San Francisco, San Francisco, USA; 3 School of Medicine, Makerere University College of Health Sciences, Kampala, Uganda; 4 London School of Hygiene and Tropical Medicine, London, UK; 5 Institute for Health Metrics and Evaluation, University of Washington, Seattle, USA; 6 Department of Medical Microbiology, Radboud University Medical Center, Nijmegen, The Netherlands

**Keywords:** Malaria, Parasitaemia, LAMP, Sub-microscopic infection

## Abstract

**Background:**

Parasite prevalence is a key metric used to quantify the burden of malaria and assess the impact of control strategies. Most published estimates of parasite prevalence are based on microscopy and likely underestimate true prevalence.

**Methods:**

Thick smear microscopy was performed in cohorts of children (aged 6 month to 10 years) and adults every 90 days over 2 years, at three sites of varying transmission intensity in Uganda. Microscopy-negative samples were tested for sub-microscopic parasitaemia using loop-mediated isothermal amplification (LAMP). Generalized estimating equation models were used to evaluate associations between age and parasitaemia, factors associated with sub-microscopic infection and associations between parasitaemia and haemoglobin.

**Results:**

A total of 9260 samples were collected from 1245 participants. Parasite prevalence among children across the three sites was 7.4, 9.4 and 28.8 % by microscopy and 21.3, 31.8 and 69.0 % by microscopy plus LAMP. Parasite prevalence among adults across the three sites was 3.1, 3.0 and 5.2 % by microscopy and 18.8, 24.2 and 53.5 % by microscopy plus LAMP. Among those with parasitaemia, adults and persons recently treated with anti-malarial therapy had the highest prevalence of sub-microscopic infection. Children with sub-microscopic or microscopic parasitaemia had lower mean haemoglobin levels compared to children with no detectable parasites.

**Conclusions:**

Across a range of transmission intensities in Uganda, microscopy vastly underestimated parasite prevalence, especially among adults.

## Background

Considerable progress has been made in the past decade in reducing the burden of malaria in Africa, largely due to interventions such as long-lasting, insecticide-treated nets (LLIN) and use of artemisinin-based combination therapy (ACT) [1]. However, accurately identifying and targeting the human reservoir of malaria parasitaemia remains a critical step to reducing the burden of malaria worldwide. This is true not only in low transmission settings approaching elimination, but also in highly endemic countries, where repeated infections cause a partial non-sterilizing immunity to malaria and a large proportion of infections may be asymptomatic.

The majority of published surveys of malaria parasite prevalence underestimate true prevalence due to the low sensitivity of microscopy and rapid diagnostic tests (RDTs), which have limited ability to detect parasitaemia <100 parasites/µL [2, 3]. Molecular techniques such as PCR are more sensitive [3], and a newer molecular technique, loop-mediated isothermal amplification (LAMP), has an estimated lower limit of detection of 1–5 parasites/µL [4]. LAMP has been used to estimate prevalence in field settings [5–7], and offers a simple tool for addressing the sub-microscopic malaria reservoir. There is an increasing awareness of implications of sub-microscopic infection, including an appreciation that sub-microscopic infections can be prominent contributors to malaria transmission [8, 9].

In order to estimate the prevalence of microscopic and sub-microscopic parasitaemia across different transmission intensities in Uganda, repeated sampling of a cohort of children and adults was performed with microscopy and LAMP in three different epidemiologic settings over 2 years. Relationships between age and prevalence of parasitaemia, as well as between parasitaemia and mean haemoglobin, were evaluated, with the hypothesis that microscopy would underestimate parasite prevalence compared to LAMP, in all age groups and epidemiologic settings and that participants with microscopic and sub-microscopic parasitaemia would have lower mean haemoglobin than those with no infection.

## Methods

### Study sites

The study was conducted in Nagongera sub-county, Tororo District in eastern Uganda, Walukuba sub-county, Jinja District in eastern Uganda and Kihihi sub-county, Kanungu District in southwestern Uganda. Study clinics were set up at health centre facilities that serve each of the sub-counties. Nagongera and Kihihi sub-counties are predominantly rural and have higher malaria transmission intensities, while Walukuba is peri-urban, with the lowest transmission intensity of the three sites [10].

### Study design and participants

Enrolment of cohorts has been described previously [10, 11]. Briefly, participants were recruited from 100 randomly selected households within the catchment area of the participating health facility at each of the three sites. All children aged 6 months to 10 years and a primary adult caretaker in a household were enrolled. As the cohort aged, all children that reached 6 months of age were dynamically enrolled and children ≥11 years of age were excluded from further follow-up. Participants agreed to come to the study clinic for any febrile illness and to avoid anti-malarial medications administered outside the study. All enrolled participants were also given a LLIN.

### Clinical follow-up and study time period

Study participants attended the clinic at enrolment and then every 90 days for routine visits. At routine visits, they received a history and physical examination and blood was obtained for haemoglobin measurement, thick blood smear and dried blood spots (DBS) on filter paper. RDTs were not performed. Participants who reported a history of fever in the previous 24 h or had a tympanic temperature ≥38.0 °C, and had a positive blood smear, were diagnosed with malaria and treated with artemether-lumefantrine. For this analysis, all routine clinic visits from 1 August, 2011 to 30 September, 2013 were included.

### Laboratory methods

Haemoglobin was estimated from finger-prick blood samples using a portable spectrophotometer. Thick blood smears were prepared with 2 % Giemsa. Light microscopy was performed by an experienced laboratory technician who was not involved in direct patient care. A second technician verified all microscopy results and a third technician resolved discrepancies, if needed.

DBS specimens were prepared by spotting whole blood onto filter paper and drying completely. LAMP testing was performed on all DBS specimens for which a participant had a negative blood smear. DNA was extracted for LAMP using Chelex, as previously described (10). LAMP was performed using Eiken Loopamp™ Malaria Pan Detection Kit reaction tubes, which contain vacuum-dried reagents specific for amplification of *Plasmodium* species mitochondrial DNA, per manufacturer’s guidelines [12]. Results of LAMP reactions were based on visual detection of fluorescence under an ultraviolet lamp. Three controls with known parasite densities were used (10 parasites/µL, 1 parasite/µL and 0 parasite/µL), as well as LAMP kit positive and negative controls. LAMP reactions were rejected and then repeated if controls did not produce their expected results.

### Data management and statistical analysis

Data were analysed using STATA (version 13; STATA Corp., College Station, TX, USA). The primary outcomes of interest were: (1) microscopic parasitaemia, defined as the proportion of routine visits with a positive blood smear, with or without fever; (2) sub-microscopic parasitaemia, defined as the proportion of routine visits with a negative blood smear and positive LAMP reaction; and, (3) any parasitaemia, defined as the proportion of routine visits with either positive blood smear or positive LAMP reaction. Generalized estimating equation models were used to estimate associations between age and parasitaemia, to assess independent predictors of sub-microscopic parasitaemia and to estimate the association between parasitaemia and mean haemoglobin, using exchangeable correlation structure, log link and robust standard error estimates. Within subject clustering was specified to account for repeated episodes of parasitaemia in participants. For models looking at the association of age with prevalence of parasitaemia, the highest prevalence group for each site was chosen as the reference group. A p value <0.05 was considered statistically significant.

## Results

### Characteristics of the study site

Characteristics of the three study sites are presented in Table 1. Entomologic data have been described previously [10]. The annual entomological inoculation rate (aEIR) was lowest in Walukuba, with 2.8 infective bites per person per year and was approximately ten-fold higher in Kihihi and 100-fold higher in Nagongera, reflecting variation in transmission intensity among the three sites.

**Table 1:** Characteristics of study sites and participants

Characteristics	Study site		
	Walukuba	Kihihi	Nagongera
Annual entomological inoculation rate	2.8	32.0	310
*Number of study participants*			
Children	283	336	311
Adults	114	95	106
*Number of female participants, n (%)*			
Children	138 (48.8 %)	171 (50.9 %)	141 (45.3 %)
Adults	107 (93.9 %)	92 (96.8 %)	98 (92.5 %)
*Age in years at the time of routine visits, mean (SD)*			
Children	5.2 (2.7)	5.6 (2.8)	5.6 (2.7)
Adults	33.7 (9.9)	39.0 (13.8)	39.1 (13.5)
Number of routine visits	2652	3372	3236
*Parasite prevalence by microscopy * (%)			
Children	7.4	9.4	28.8
Adults	3.1	3.0	5.2
*Parasite prevalence by microscopy* *+* *LAMP* (%)			
Children	21.3	31.8	69.0
Adults	18.8	24.2	53.5

In total, 1245 participants were enrolled, with roughly equivalent proportions of child and adult participants at each site and similar mean age and gender at each site. Greater than 90 % of adult participants at all sites were female, because most adult primary caregivers enrolled from each household were female. Participants visited the clinic for routine evaluation every 90 days, contributing 2652, 3372 and 3236 routine visits in Walukuba, Kihihi and Nagongera, respectively.

Consistent with aEIR findings, parasite prevalence varied between sites (Table 1). Parasite prevalence detected by microscopy was 7.4, 9.4 and 28.8 % among children in Walukuba, Kihihi and Nagongera, respectively (p = 0.02 for comparison between Walukuba and Kihihi, p < 0.001 for other pair-wise comparisons) and 3.1, 3.0 and 5.2 % among adults (p = 0.04 for Walukuba versus Nagongera, p = 0.03 for Kihihi versus Nagongera, p = 0.90 for Walukuba versus Kihihi). When both microscopy and LAMP were used to detect parasitaemia, parasite prevalence was much higher than when only microscopy was used, with a prevalence of 21.3, 31.8 and 69.0 % for children (p < 0.001 for all pair-wise comparisons between sites) and 18.8, 24.2 and 53.5 % for adults (p = 0.01 for Walukuba versus Kihihi, p < 0.001 for all other pair-wise comparisons).

### Age trends of parasitaemia

The relationship between age and the prevalence of microscopic parasitaemia varied depending on transmission intensity, although at all sites prevalence increased from infancy to later in childhood and declined in adults (Table 2). In Walukuba, the lowest transmission setting, the prevalence of microscopic parasitaemia was lowest in the 6 months to <2 years age group and was significantly lower among individuals ages 6 months to <2 years and ≥18 years compared to those aged 2 to <5 years. However, in the higher transmission settings of Kihihi and Nagongera, prevalence of microscopic parasitaemia was lowest among adults. This was particularly striking in Nagongera, with a prevalence of 16.0 % among 6 months to <2 years olds versus a prevalence of 5.2 % among adults (p < 0.001). Both 6 months to <2 years olds and ≥18 year olds in Kihihi and Nagongera had a significantly lower prevalence of microscopic parasitaemia than a reference group of 5 to <11 years olds.

**Table 2:** Associations between age and prevalence of microscopic parasitaemia, stratified by study site

Age group (years)	Walukuba			Kihihi			Nagongera		
	Prevalence	RR (95 % CI)	p value	Prevalence	RR (95 % CI)	p value	Prevalence	RR (95 % CI)	p value
0.5– <2	6/247 (2.4 %)	0.36 (0.17–0.74)	0.005	14/294 (4.8 %)	0.43 (0.23–0.80)	0.007	42/263 (16.0 %)	0.51 (0.37–0.71)	<0.001
2– <5	56/666 (8.4 %)	Reference group		71/843 (8.4 %)	0.76 (0.56–1.05)	0.093	189/740 (25.5 %)	0.77 (0.65–0.91)	0.002
5– <11	74/936 (7.9 %)	0.90 (0.58–1.40) 0.637		159/1470 (10.8 %)	Reference group		456/1380 (33.0 %)	Reference group	
≥18	25/803 (3.1 %)	0.37 (0.21–0.64)	<0.001	23/765 (3.0 %)	0.28 (0.17–0.45)	<0.001	44/853 (5.2 %)	0.15 (0.11–0.22)	<0.001

Relative risk is adjusted for repeated measures in the same study participant

Reference group is defined as the group with highest prevalence, at all sites

The relationship between age and parasitaemia was slightly different when considering any parasitaemia, including both microscopic and sub-microscopic infection (Table 3). In Walukuba, the lowest transmission site, the age trend was similar to that of microscopic parasitaemia. Children 6 months to <2 years had a significantly lower prevalence of parasitaemia, compared to the 2 to <5 years reference group, who had the highest prevalence of parasitaemia; the prevalence of parasitaemia declined for older children and adults, but these relative risks were not significant. At the higher transmission sites of Kihihi and Nagongera, the prevalence of parasitaemia was also lowest among children 6 months to <2 years. In contrast to Walukuba, however, the prevalence of any detectable parasitaemia among adults in Kihihi and Nagongera did not follow the same pattern as the microscopy data. Instead of adults being the lowest prevalence group, as was true for microscopic parasitaemia, when both LAMP and microscopy were considered, adults had a similar prevalence of parasitaemia to that of infants.

**Table 3:** Associations between age and prevalence of any parasitaemia, stratified by study site

Age group (years)	Walukuba			Kihihi			Nagongera		
	Prevalence	RR (95 % CI)	p value	Prevalence	RR (95 % CI)	p value	Prevalence	RR (95 % CI)	p value
0.5– <2	31/247 (12.6 %)	0.62 (0.44–0.88)	0.008	52/294 (17.7 %)	0.55 (0.40–0.76)	<0.001	129/263 (49.1 %)	0.66 (0.58–0.75)	<0.001
2– <5	164/666 (24.6 %)	Reference group	–	229/843 (27.2 %)	0.82 (0.67–0.99)	0.04	471/740 (63.7 %)	0.85 (0.79–0.92)	<0.001
5– <11	198/936 (21.2 %)	0.79 (0.62–1.02) 0.071		548/1470 (37.3 %)	Reference group		1045/1380 (75.7 %)	Reference group	
≥18	151/803 (18.8 %)	0.76 (0.57–1.01)	0.06	185/765 (24.2 %)	0.66 (0.51–0.85)	0.001	456/853 (53.5 %)	0.71 (0.64–0.78)	<0.001

Relative risk is adjusted for repeated measures in the same study participant

Reference group is defined as the group with highest prevalence, at all sites

### Factors associated with sub-microscopic infection, among those with any parasitaemia

**Fig. 1:**
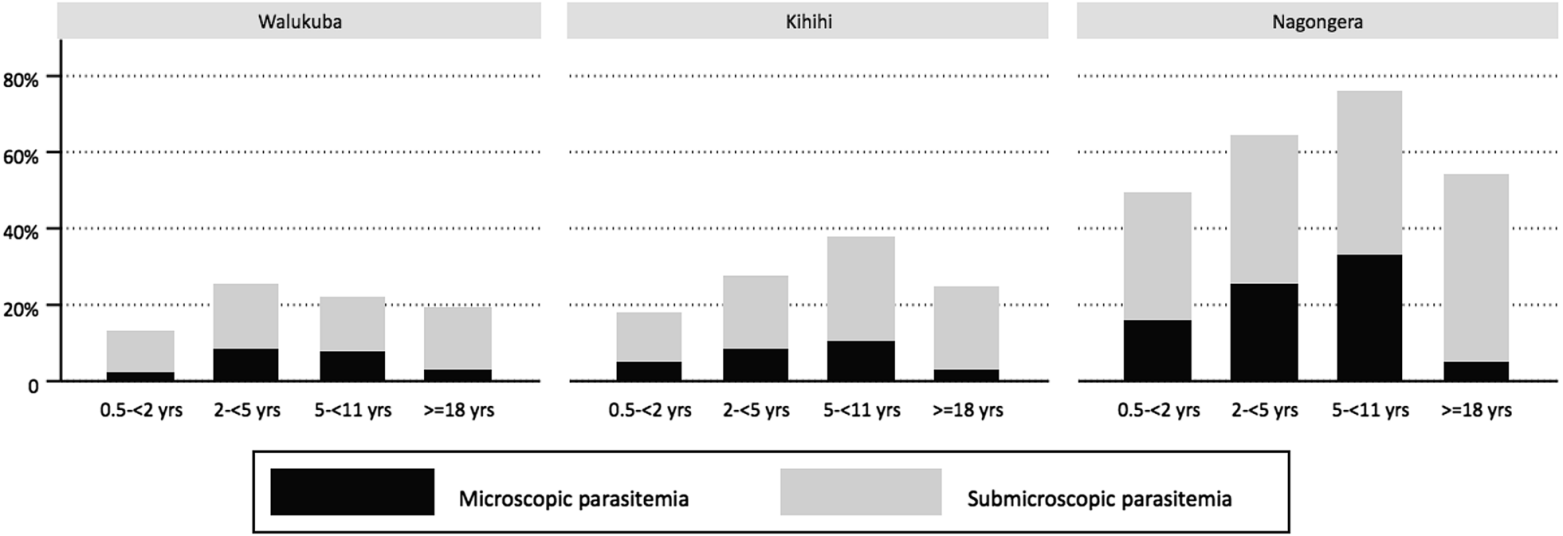
Proportion of participants with sub-microscopic parasitaemia and microscopic parasitaemia, by site. Participants are divided into sites and age strata along the *X*-axis. The proportion of positive samples, among all tested samples for that age and site, are shown on the *Y*-axis

The majority of parasitaemia detected in this study was sub-microscopic, across all ages and settings (Fig. 1). Among children with any parasitaemia, the proportion of infections that were sub-microscopic was highest in Kihihi at 69.7 %, compared to 61.5 % in Walukuba and 59.1 % in Nangongera (p = 0.01 for Walukuba versus Kihihi, p < 0.001 for Kihihi versus Nagongera, p = 0.44 for Walukuba versus Nagongera). Among parasitaemic adults, the only significant difference between sites in the proportion of sub-microscopic infections was between Walukuba and Nagongera (83.2 versus 90.1 %, p = 0.04).

Among parasitaemic children, there was no significant association demonstrated between sub-microscopic parasitaemia and age category (Table 4). Compared to a reference group aged 5 to <11 years, parasitaemic adults were more likely to have sub-microscopic infection, at all sites. The effect of age ≥18 years on the prevalence of sub-microscopic parasitaemia was most dramatic in the medium- and highest-transmission sites, with >90 % of all detectable infections in Nagongera adults due to sub-microscopic parasitaemia. Compared to those not recently treated, a history of being diagnosed and treated for malaria in the past 30 days was associated with a significantly greater prevalence of sub-microscopic parasitaemia among those who were parasitaemic, at all sites.

**Table 4:** Factors associated with sub-microscopic parasitaemia if parasitaemic, stratified by study site

Factor	Walukuba			Kihihi			Nagongera		
	Prevalence	RR (95 % CI)	p value	Prevalence	RR (95 % CI)	p value	Prevalence	RR (95 % CI)	p value
*Age groups* (years)									
0.5– <2	14/18 (77.8 %)	1.22 (0.94–1.58)	0.14	23/34 (67.7 %)	0.95 (0.75–1.22)	0.71	70/97 (72.2 %)	1.11 (0.96–1.29)	0.15
2– <5	81/126 (64.3 %)	1.09 (0.89–1.33)	0.41	146/206 (70.9 %)	1.00 (0.90–1.12)	0.95	243/401 (60.6 %)	1.02 (0.91–1.13)	0.77
5– <11	95/165 (57.6 %)	Reference group		336/485 (69.3 %)	Reference group		533/933 (57.1 %)	Reference group	
≥18	99/119 (83.2 %)	1.43 (1.20–1.71)	<0.001	140/163 (85.9 %)	1.25 (1.13–1.38)	<0.001	354/393 (90.1 %)	1.65 (1.53–1.79)	<0.001
*Symptomatic malaria in prior 30* *days*									
No	263/399 (65.9 %)	Reference group		537/761 (70.6 %)	Reference group		987/1562 (63.2 %)	Reference group	
Yes	26/29 (89.7 %)	1.36 (1.17–1.58)	<0.001	108/127 (85.0 %)	1.27 (1.15–1.40)	<0.001	213/262 (81.3 %)	1.42 (1.31–1.55)	<0.001

Relative risk is adjusted for repeated measures in the same study participant

Excludes samples from enrollment visits, for which data on malaria diagnosis in past 30 days is missing

### Associations between parasitaemia status and haemoglobin

Among children in all sites, mean haemoglobin was significantly lower in those with any parasitaemia, compared to the group with no detectable parasitaemia (Table 5). There was a step-wise decrease in mean haemoglobin in children from microscopic to sub-microscopic to no detectable infection. These differences remained significant when participants with febrile episodes of malaria were excluded from the analysis. For adults, a similar trend was demonstrated in Walukuba and Kihihi, with a significantly lower mean haemoglobin demonstrated among those with microscopic parasitaemia in Walukuba and a lower mean haemoglobin in those with sub-microscopic parasitaemia and microscopic parasitaemia in Kihihi. In Nagongera, the highest transmission site, there was no significant difference in mean haemoglobin among adults, regardless of parasitaemia status.

**Table 5:** Associations between parasitaemia status and mean haemoglobin, stratified by age and study site

Parasitemia status	Walukuba			Kihihi			Nagongera		
	Haemoglobin mean (SD)	Relative change (95 % CI)	p value	Haemoglobin mean (SD)	Relative change (95 % CI)	p value	Haemoglobin mean (SD)	Relative change(95 % CI)	p value
*Children*									
No detectable parasitaemia	12.0 (1.4)	Reference group		12.7 (1.5)	Reference group		11.9 (1.4)	Reference group	
Sub-microscopic parasitaemia	11.4 (1.7)	0.96 (0.94–0.97)	<0.001	12.0 (1.6)	0.94 (0.93–0.95)	<0.001	11.6 (1.5)	0.97 (0.96–0.98)	<0.001
Microscopic parasitaemia	10.9 (1.7)	0.91 (0.89–0.93)	<0.001	11.6 (1.6)	0.91 (0.90–0.93)	<0.001	11.4 (1.5)	0.95 (0.93–0.96)	<0.001
*Adults*									
No detectable parasitaemia	13.3 (1.6)	Reference group		13.6 (1.8)	Reference group		13.3 (1.6)	Reference group	
Sub-microscopic parasitaemia	13.0 (1.8)	0.98 (0.97–1.00)	0.13	12.8 (1.6)	0.95 (0.93–0.98)	<0.001	13.2 (1.7)	0.99 (0.98–1.01)	0.20
Microscopic parasitaemia	12.9 (1.8)	0.95 (0.91–0.99)	0.008	12.4 (2.2)	0.93 (0.88–0.98)	0.01	13.5 (1.6)	0.99 (0.96–1.02)	0.46

Relative change is adjusted for age and repeated measures in the same study participant

## Discussion

Presented in this report is the prevalence of microscopic and sub-microscopic parasitaemia in three cohorts with varied transmission intensity in Uganda. A highly sensitive diagnostic test was used to characterize parasite prevalence as it relates to age, transmission intensity and recent anti-malarial treatment. As seen by others [2–4, 13], microscopy dramatically underestimated malaria parasite prevalence. Compared to microscopy, LAMP increased the proportion of samples with detectable parasitaemia two- to ten-fold, depending on age and site. This study clarifies the age-specific trends of sub-microscopic parasitaemia, allows these trends to be directly compared to those of microscopic parasitaemia and identifies factors associated with sub-microscopic infection. In those with detectable parasitaemia, adulthood and recent treatment were associated with increased prevalence of sub-microscopic infection. Children with either sub-microscopic or microscopic parasitaemia had lower mean haemoglobin than children with no detectable parasites across study sites, demonstrating the clinical relevance of sub-microscopic infection.

The majority of published survey data rely on microscopy or RDT to generate estimates of malaria parasite prevalence. These surveys underestimate the true prevalence of parasitaemia since they do not detect low density infection [3]. Prior studies suggest that the proportion of sub-microscopic infections varies with transmission intensity and that sub-microscopic infections may predominate in low-transmission settings [14]. This study suggests that sub-microscopic infections make up the majority of detectable parasitaemia across all ages and transmission intensities in Uganda. Despite a dramatically different overall prevalence of parasitaemia at the three study sites, the proportion of sub-microscopic infection among parasitaemic participants (59–70 % in children, 83–90 % in adults) was not only substantial, but also remarkably consistent across transmission intensities. Indeed, it is possible that due to the lower limit of detection of LAMP compared to more sensitive PCR-based assays, this study underestimated the size of the parasite reservoir, particularly among adults, in whom the majority of infections were sub-microscopic and mean parasite densities were likely the lowest.

These data demonstrate that the prevalence of parasitaemia increases as children age and then declines in adulthood. This is a well described trend in malaria epidemiology [15], and consistent with previously published reports in this region [10, 11, 16]. However, differences in age trends emerged when sub-microscopic parasitaemia was considered in addition to microscopic parasitaemia. In contrast to microscopic parasitaemia, for which adults had the lowest prevalence in Kihihi and Nagongera, the prevalence of any parasitaemia was lowest at all sites in the youngest age group, aged 6 months to <2 years. Published prevalence estimates for children of this age usually reflect the care of infants admitted to tertiary care facilities [17], or are reported combined with older age groups [18]. Passive immunity related to maternal antibodies is thought to wane between 6 and 12 months [19, 20], but the nadir prevalence of parasitaemia in children <2 years may be related to force of infection related to low body surface area [21], increased use of LLINs in this age group [22], or the fact young children are more likely to become symptomatic and be treated for malaria.

Across all sites, the change in parasite prevalence with sub-microscopic detection of parasites was most striking in adults, with the prevalence of parasitaemia six- to ten-fold higher when considering LAMP results. In the highest transmission setting, Nagongera, the addition of sub-microscopic data for persons ≥18 years old increased the prevalence of parasitaemia from 5 to over 50 %, giving adults a prevalence similar to that of infants. This study demonstrates that among parasitaemic individuals there is a significant association between age ≥18 years and prevalence of sub-microscopic parasitaemia. This finding is consistent with a recently published review of malaria diagnostics, which describes adults as having the highest proportion of subpatent infections, regardless of transmission intensity [3]. Adults, if parasitaemic, are more likely to have lower density infections, likely related to acquired partial immunity in adulthood and the ability of the adult immune system to suppress blood-stage parasites [23, 24].

Being diagnosed and treated for malaria in the previous 30 days was associated with a greater prevalence of sub-microscopic parasitaemia among those who were parasitaemic. This effect reflects the sensitivity of LAMP as a diagnostic test and may be due to residual low-density asexual parasitaemia, persistence of mature gametocytes following treatment, amplification of DNA from non-viable parasites, or suppression of recurrent parasite infection by lumefantrine. The phenomenon of residual sub-microscopic parasitaemia and gametocytaemia following treatment with ACT has been previously described [25, 26], but the transmission potential of recently treated individuals remains an area that is poorly understood.

Both sub-microscopic and microscopic parasitaemia are associated with reduced mean haemoglobin, as supported by the results of this study. The association between acute episodes of malaria and anaemia is well described, with cumulative episodes of haemolysis leading to impairments in red cell production [27, 28]. The association between parasitaemia and anaemia is less clear, although it has been described in children with both microscopic and sub-microscopic parasitaemia [29, 30]. The results of this study demonstrate significantly lower mean haemoglobin in children with both sub-microscopic and microscopic parasitaemia, compared to the group with no detectable parasitaemia. Adults showed a similar trend in lower transmission sites, although this pattern was not observed in highest transmission site. Anaemia is multifactorial and the analysis of this study did not take into account factors such as genetic polymorphisms, nutrition, parity, or helminth infection, nor did it assess cumulative effects of malaria on an individual. Furthermore, the study design does not allow evaluation of causality between parasitaemia and anaemia. However, this study joins a body of work suggesting deleterious clinical consequences of parasitaemia of any level and suggests that the term ‘asymptomatic infection’ may be a misnomer [31, 32].

This study had several limitations. Although the results demonstrate that people with sub-microscopic infection make up a large part of the parasite reservoir, the transmission potential of these individuals is unclear. There are data supporting mosquito infectivity of sub-microscopic parasitaemia [8], however further work is needed to clarify the relative contribution of sub-microscopic infections to onward transmission. Given the complex interplay of host factors and repeated parasitaemia, longitudinal analysis is needed to characterize the effect of parasitaemia on clinical outcomes, including anaemia. Finally, there is no way to differentiate between chronic and repeated infections in this study. Given the likelihood of multiple co-infecting strains of malaria, as well as data that suggest infections may be harboured in individuals for extended periods of time [33, 34], genotyping in future studies would be helpful and enable a better understanding of infection dynamics.

## Conclusion

This study demonstrates that the majority of individuals with detectable malaria parasitaemia have sub-microscopic infection, among all ages and across a variety of transmission intensities in Uganda. Among those with parasitaemia, adults and persons recently treated with anti-malarial therapy had the highest prevalence of sub-microscopic infection. These data should inform the discussion of malaria control efforts in high-transmission settings, where addressing sub-microscopic parasitaemia is needed to make meaningful progress towards improved malaria control and eventual elimination.

## Abbreviations

ACT: artemisinin-based combination therapy
aEIR: annual entomological inoculation rate
DBS: dried blood spot
LAMP: loop-mediated isothermal amplification
LLIN: long-lasting insecticide treated nets
PCR: polymerase chain reaction
RDT: rapid diagnostic test
